# Identifying *Pseudomonas syringae* Type III Secreted Effector Function via a Yeast Genomic Screen

**DOI:** 10.1534/g3.118.200877

**Published:** 2018-12-20

**Authors:** Amy Huei-Yi Lee, D. Patrick Bastedo, Ji-Young Youn, Timothy Lo, Maggie A. Middleton, Inga Kireeva, Jee Yeon Lee, Sara Sharifpoor, Anastasia Baryshnikova, Jianfeng Zhang, Pauline W. Wang, Sergio G. Peisajovich, Michael Constanzo, Brenda J. Andrews, Charles M. Boone, Darrell Desveaux, David S. Guttman

**Affiliations:** * Department of Cell & Systems Biology, University of Toronto, Toronto, Ontario, Canada; † The Donnelly Centre, University of Toronto, Toronto, Ontario, Canada; ‡ Centre for the Analysis of Genome Evolution & Function, University of Toronto, Toronto, Ontario, Canada; § Lewis-Sigler Institute for Integrative Genomics, Princeton University, Princeton, New Jersey; ** Department of Molecular Genetics, University of Toronto, Toronto, Ontario, Canada

**Keywords:** *Pseudomonas syringae*, Type III secreted effector, Pathogenic Genetic Array, Yeast screen, Pathogen-host interactions, HopZ1, Kinesin

## Abstract

Gram-negative bacterial pathogens inject type III secreted effectors (T3SEs) directly into host cells to promote pathogen fitness by manipulating host cellular processes. Despite their crucial role in promoting virulence, relatively few T3SEs have well-characterized enzymatic activities or host targets. This is in part due to functional redundancy within pathogen T3SE repertoires as well as the promiscuity of individual T3SEs that can have multiple host targets. To overcome these challenges, we generated and characterized a collection of yeast strains stably expressing 75 T3SE constructs from the plant pathogen *Pseudomonas syringae*. This collection is devised to facilitate heterologous genetic screens in yeast, a non-host organism, to identify T3SEs that target conserved eukaryotic processes. Among 75 T3SEs tested, we identified 16 that inhibited yeast growth on rich media and eight that inhibited growth on stress-inducing media. We utilized Pathogenic Genetic Array (PGA) screens to identify potential host targets of *P. syringae* T3SEs. We focused on the acetyltransferase, HopZ1a, which interacts with plant tubulin and alters microtubule networks. To uncover putative HopZ1a host targets, we identified yeast genes with genetic interaction profiles most similar (*i.e.*, congruent) to the PGA profile of HopZ1a and performed a functional enrichment analysis of these HopZ1a-congruent genes. We compared the congruence analyses above to previously described HopZ physical interaction datasets and identified kinesins as potential HopZ1a targets. Finally, we demonstrated that HopZ1a can target kinesins by acetylating the plant kinesins HINKEL and MKRP1, illustrating the utility of our T3SE-expressing yeast library to characterize T3SE functions.

Bacterial pathogens of both plants and animals subvert key host processes in order to suppress host immunity and manipulate nutrient supplies. Many Gram-negative bacterial pathogens achieve this goal by delivering type III secreted effectors (T3SEs) into the host cytosol where they manipulate the host in a variety of ways, including modulating signaling pathways, transcription, intracellular transport, cytoskeletal stability, and host defenses (Büttner and Bonas 2003; Jin *et al.* 2003; Cornelis 2006; Zhou and Chai 2008; Lewis *et al.* 2009). Although many bacterial T3SEs have been shown to generally suppress host immunity, we know relatively little about the specific virulence targets and mechanisms of action of most T3SEs. The difficulty in functional characterization of T3SE virulence mechanisms is due to a number of factors, including: (1) redundant targeting of a given host protein by multiple effectors which confounds analysis of individual T3SE deletion mutants; (2) promiscuous individual effectors which can target multiple host proteins, thereby making it difficult to ascribe a virulence function to any individual target (Lewis *et al.* 2011; Deslandes and Rivas 2012); (3) effectors often show no similarity to proteins or domains with characterized functions, limiting bioinformatic approaches to infer effector functions; and (4) effectors can trigger immune responses as a result of host recognition, which complicates virulence target identification.

In order to gain new insights into the biochemical functions and host targets of bacterial T3SEs, a number of research groups have utilized the model organism *Saccharomyces cerevisiae* (yeast) as a tool (Yoon *et al.* 2003; Jamir *et al.* 2004; Alto *et al.* 2006; Kramer *et al.* 2007; Slagowski *et al.* 2008; Alemán *et al.* 2009; Munkvold *et al.* 2009; Salomon and Sessa 2010). The rationale for using yeast to characterize bacterial effectors rests on the fact that many biological processes (for example central metabolism, the control of cytoskeleton dynamics, vesicle trafficking, signal transduction, DNA metabolism and cell cycle processes) are conserved among eukaryotes (Dolinski and Botstein 2007; Siggers and Lesser 2008; Curak *et al.* 2009; Botstein and Fink 2011). Therefore, effectors that target a conserved cellular process in a higher eukaryote may also act on the same cellular process in the simpler and genetically-tractable yeast system. This is particularly attractive if the original host is not readily amenable to high-throughput assays. Another advantage of studying bacterial T3SEs in the yeast system is that the expression of non-effector bacterial proteins does not generally affect yeast growth (Slagowski *et al.* 2008). This indicates that most fitness defects observed upon T3SE expression in yeast is specifically due to T3SE activity, and not simply due to the heterologous overexpression of bacterial proteins. Finally, the expression of translocated effector proteins from both plant and animal pathogens has been shown to inhibit yeast growth by targeting conserved eukaryotic proteins (Munkvold *et al.* 2008; Siggers and Lesser 2008; Curak *et al.* 2009; Salomon *et al.* 2011). For instance, the *Yersinia* T3SE YopJ has been shown to disrupt mammalian innate immunity by preventing the activation of MAPK kinase (MAPKK) and subsequently blocking the MAPK and NFκB signaling pathways (Orth *et al.* 1999; Orth *et al.* 2000). Even though yeast cells lack key components of the mammalian innate immune system, YopJ was shown to inhibit MAPK pathways in yeast by preventing the activation of MAPKK as previously observed in mammalian systems (Yoon *et al.* 2003).

A number of groups have developed yeast genomics tools to characterize bacterial effectors that target conserved eukaryotic cellular processes (Alto *et al.* 2006; Kramer *et al.* 2007). A very successful genetic approach is the Pathogenic Genetic Array (PGA), a variation of the well-established Synthetic Genetic Array (SGA) technology, which enables high-throughput genetic screens to identify conserved host targets (Alto *et al.* 2006; Kramer *et al.* 2007). The SGA technology involves a series of robotics-assisted cell matings to introduce any marked allele of interest into an array of mutants, allowing the systematic generation of double mutants and the interrogation of di-genic genetic interactions at a genome-wide scale (Tong *et al.* 2001; Tong *et al.* 2004; Costanzo *et al.* 2010). Genetic interactions between two mutations are inferred when the observed double mutant phenotype deviates from the expected phenotype of the combined single mutants. In extreme cases, a synthetic lethal interaction occurs when the combination of two non-lethal mutations causes cell death. Large scale, genome-wide SGA screens have provided global genetic interaction profiles in the yeast genome (Costanzo *et al.* 2010; Costanzo *et al.* 2016). Since genes within the same pathway or bioprocess tend to show very similar genetic interaction profiles, querying the genetic interactions of an unknown gene against the nearly complete SGA compendium of the yeast genome can be a powerful way to predict functions of uncharacterized genes (Costanzo *et al.* 2010).

Similar to SGA, PGA queries a pathogen effector against a collection of viable yeast deletion strains in a high-throughput array format to analyze effector functions. PGA identifies those yeast deletion mutants that interact genetically with T3SE, assessed by fitness of combined mutants showing greater or lower fitness than expected, and subsequently guides the inference of functional relationships between these yeast genes and the pathogen T3SEs (Alto *et al.* 2006; Kramer *et al.* 2007). This PGA strategy was first used to identify yeast deletion mutants that suppress *Shigella* T3SE IpgB2-induced toxicity (Alto *et al.* 2006). Consistent with the ability of IpgB2 to interfere with Rho1p signaling in mammalian cells, the genetic suppressors of IpgB2 in yeast are downstream of Rho1p, part of the cell wall integrity MAPK-signaling pathway (Alto *et al.* 2006). Overall this PGA screen revealed that IpgB2 functions as a G protein mimic, capable of activating the Rho1p pathway (Alto *et al.* 2006).

In this study, we hypothesized that T3SEs that target evolutionarily conserved plant processes can regulate the same processes in yeast. Furthermore, if this conserved process is important for optimal yeast growth, then the overexpression of T3SEs should decrease yeast fitness. We generated a library of 75 *P. syringae* T3SE-expressing yeast strains and identified 24 effectors that reduced yeast fitness in either standard rich media or under high osmotic stress. We performed PGA screens on five T3SEs and established genetic interaction profiles for three: HopF2*_PtoT1_*, HopX1*_PmaES4326_* and HopZ1a*_PsyA2_*. We used HopZ1a as our proof-of-principle T3SE, and compared the genetic interaction profile of HopZ1a with previously generated SGA datasets (Costanzo *et al.* 2010) to identify yeast genes with interaction profiles similar (or congruent) to that of HopZ1a in order to identify potential HopZ1a targets. Among the yeast genes with interaction profiles congruent to HopZ1a were kinesins, which have been previously shown to physically interact with HopZ1a (Mukhtar *et al.* 2011; Lewis *et al.* 2012). These findings implicate kinesins as putative targets of HopZ1a. In support of this, we have demonstrated that HopZ1a can acetylate *Arabidopsis thaliana* (hereafter *Arabidopsis*) kinesin proteins. This study emphasizes the power of high-throughput heterologous screens for exploration of T3SE function and for identification of conserved eukaryotic processes that are targeted by diverse pathogens.

## Materials and Methods

### Cloning

Promoter-less coding sequences lacking stop codons of *P. syringae* T3SEs were PCR-amplified to include the addition of *att*B1 and *att*B2 linkers and cloned into the Gateway donor vector, pDONR207, using the Gateway BP reactions. T3SEs from *Pto*DC3000, *Psy*B728a and *Pph*1448a were generous gifts from J. Chang (Chang *et al.* 2005). The additional T3SEs from *Pma*ES4326, as well as T3SEs from the HopZ and HopF families were cloned for this study. The pDONR207-T3SE collection was sequenced-confirmed via Sanger sequencing. These T3SEs were subcloned into the Gateway-compatible yeast integration vector, pBA2262 (Youn *et al.* 2017), using the Gateway LR reactions. To confirm the pBA2262-T3SE constructs, purified plasmids were digested with *Bsr*GI or *Not*I and the restriction digest patterns were analyzed.

Promoter-less coding sequences of *A. thaliana* kinesins *HINKEL* (At1g18370) and *MKRP1* (At1g21730) lacking stop codons were likewise PCR-amplified and cloned into pDONR207 and were subcloned by Gateway LR reactions into the autonomously-replicating, single-copy, Gateway-compatible yeast expression vector, pBA350V (Lewis *et al.* 2013; Youn *et al.* 2017).

### Yeast strain construction, growth medium, immunoblot analyses

To integrate the P*_GAL1_-T3SE-FLAG*::*NAT^R^* constructs into the yeast genome at the *ho* locus, the SGA query strain (Y7092, MATα, *can1Δ*::*STE2pr-Sp_his5 lyp1Δ his3Δ1 leu2Δ0 ura3Δ0 met15Δ0*) was transformed with *Not*I-digested BA2262-T3SE plasmid DNA using the standard transformation method (Gietz and Woods 2002).

For immunoblot analyses, yeast strains expressing the FLAG-tagged T3SEs under control of the *GAL1* promoter were grown overnight at 30° shaking (200 RPM) in 1 ml of YP broth with 2% raffinose (YPR) in deep-well plates with sterile glass beads in each well. The overnight cultures were subsequently diluted into deep-well plates containing 1 ml of YP broth with 2% galactose (YPG) at OD_600_ of 0.1. The cultures were induced for T3SE expression for 7 to 8 hr, or until the cultures reach OD_600_ of 1. The 1 ml-cultures were pelleted at 13,000 x *g* for 1 min, washed, and frozen at -20°. Whole cell extracts were prepared from trichloroacetic acid (TCA)-fixed cells as described (Kurat *et al.* 2009). The protein pellets were resuspended in 1X sample buffer and neutralized by addition of 2M Tris solution. The lysates were separated by 12% SDS-PAGE and immunoblot was performed with mouse anti-FLAG primary antibodies (Sigma, F3165, USA) via chemiluminescence (Amersham, USA).

### Pathogenic genetic array

The pathogenic genetic array (PGA) analysis was based on a variation of the SGA method used for synthetic dosage lethality screens (Tong *et al.* 2001; Sopko *et al.* 2006). In brief, Y7092 (the SGA query strain) with integrated *hoΔ*::*GAL1-T3SE-FLAG*::*NAT^R^* was mated into the 1536-density *MATa* deletion mutant array marked with *KAN^R^*, which represents each single mutant colony four times on the array. Y7092 carrying *hoΔ*::*NAT^R^* (SN851) was used as a negative control strain. The *MATa/α* diploids were selected on YPD supplemented with clonNAT (100 μg/ml) and G418 (200 μg/ml) at 30° for two days. Diploid cells were pinned onto enriched sporulation media (20 g/L agar, 10 g/L potassium acetate, 1 g/L yeast extract, 0.5 g/L glucose, 0.1 g/L amino acids-supplement) and allowed to sporulate at 22° for at least one week. The spores were pinned onto synthetic dextrose (SD) media (Tong *et al.* 2004) – His/Arg/Lys + clonNAT/canavanine/thialysine and incubated at 30° for two days to select for *MATa* haploid meiotic progeny. The drugs canavanine and thialysine were used at 50 μg/ml. The *MATa* haploid meiotic progeny were subsequently pinned onto SD – His/Arg/Lys + clonNAT/ canavanine/ thialysine/ G418 plates twice to select for the final *MATa* meiotic progeny carrying both the *kan^R^* (yeast deletion strains) and *NAT^R^* (*GAL1-T3SE-FLAG* constructs) markers. To induce for T3SE expression, the *MATa* haploid meiotic progeny from final selection were pinned onto the synthetic galactose (SG) media – His/Arg/Lys + clonNAT/canavanine/thialysine/G418, and in the case of the HopZ1a screen the plates also contain 0.5M NaCl, followed by incubation of plates at 30° for two day.

In order to generate double mutants successfully using the SGA procedure, each array plate of haploid deletion strains contained a border of wild type yeast carrying the necessary selectable markers to correct for edge effects, where colonies toward the edge of the plate have greater access to nutrients and are therefore larger in size compared to colonies near the center of the plate (Baryshnikova *et al.* 2010; Wagih *et al.* 2013). Lastly, to ensure that the expression of effectors did not inhibit yeast mating or sporulation, all of the strain construction steps utilized glucose-containing media to repress effector expression.

After obtaining images of final plates, we quantified colony sizes and assessed fitness manually. In detail, we assessed the fitness of double mutants relative to the single mutants by comparing the colony size of each mutant on the experiment array (T3SE-expression combined with a yeast gene deletion; Figure 2C bottom panel) and the control array (no T3SE, fitness of yeast deletion mutant only; Figure 2C top panel), all on T3SE-expressing (galactose) media. We were able to indirectly assess T3SE-associated fitness by gauging the overall fitness of all the strains in the experimental plate.

### Confirmation of PGA interactors

Yeast deletion strains that were either putative suppressors or synthetic lethal interactors from the PGA screens were streaked out on YPD with 200 μg/ml of G418 (Invitrogen Life Technologies, USA) and incubated at 30° for 2 – 3 days. Single colonies of each deletion strain were patched onto YPD plates in 1 – 2 cm^2^ patches and incubated at 30° for 1 overnight to allow for actively growing yeast cultures. A single colony of wild type yeast from the deletion array border was also streaked out and patched onto YPD plates as control strains. Each yeast deletion strain was scraped off from the patches (∼10^8^ – 10^9^ cells) using sterile toothpicks and arrayed into a 96-well microtiter plate containing 200 μl of sterile water. Yeast cells were washed once with 200 μl of 0.1 M lithium acetate by centrifugation for 5 min at 1,500 x *g* at 20° in a centrifuge with a microtiter plate rotor. Each well of pelleted yeast cells was resuspended with 180 μl of transformation mix (120 μl of 50% w/v PEG-3350, 18 μl of 1 M lithium acetate, and 25 μl of boiled single-stranded carrier DNA). 60 μl each of resuspended cells were subsequently transferred to 96-well microtiter plates containing either 1 μl of purified plasmid DNA pBA350V (empty vector) (Lewis *et al.* 2013; Youn *et al.* 2017) or 1 μl of purified plasmid DNA (pBA350V*-hopZ1a*, pBA350V*-hopF2* and pBA350V*-hopX1*). The remaining 60 μl of cells served as a mock transformation control. The 96-well microtiter plates were incubated at 30° for 30 min followed by heat shock at 42° for 30 min. Cells were harvested by centrifugation for 10 min at 1,500 x *g* at 20° and resuspended in 100 μl of SD. 50 μl of transformed or mock-transformed cells were plated on SD-Leu and incubated at 30° for 3 days. Transformants carrying either pBA350V or pBA350V-T3SE (pBA350V*-hopZ1a*, pBA350V*-hopF2* and pBA350V*-hopX1*) were grown on SD-Leu plates and were subsequently used for confirmation by spot dilution assays. In order to confirm positive or negative genetic interactions, we used the number of spots to calculate the fitness of each single or double mutant in semi-quantitative manner, as described in (Youn *et al.* 2017).

### Spot dilution assay

For spot dilution assay to determine growth inhibition of Y7092 expressing *P. syringae* T3SEs, 1 ml of cultures were grown at 30° and 200 RPM in YPR in deep-well plates that contain sterile glass beads in each well. Ten-fold dilution series of the overnight cultures were spotted onto YPD, YPG, YPD with 1 M sorbitol, YPG with 1 M sorbitol, YPD with 1 M NaCl, or YPG with 1 M NaCl.

For spot dilution assays to confirm the putative PGA hits as either suppressors or synthetic lethal interactors, the deletion strains carrying either the empty vector (pBA350V) or the effector of interest (pBA350V*-T3SE)* were grown in synthetic drop-out media lacking Leu with 2% raffinose (SR-Leu) for two overnights at 30° and 200 RPM. The overnight cultures were serially diluted 15-fold and spotted onto SD-Leu, SG-Leu, SD-Leu and 0.5 M NaCl, or SG-Leu and 0.5 M NaCl. Spot dilutions were grown for two to three days before being photographed. Spot assays were quantified using an unbiased visual toxicity score (between 1 to 5), where 1 represented the strongest toxicity (1 spot grew) and 5 represented the least toxicity (all 5 spots grew). A fitness defect score was subsequently calculated using the toxicity score to compare the expected fitness defect to the observed fitness defect of each mutant (Baryshnikova *et al.* 2010; Sharifpoor *et al.* 2012).

### Gene Ontology (GO) Enrichment Analysis

GO enrichment analysis was performed by entering query genes (either HopZ1a PGA interactors or yeast mutants with congruent SGA interaction profiles as HopZ1a) into the GO Term Finder of the Saccharomyces Genome Database (https://www.yeastgenome.org/goTermFinder) using a gene universe (background gene set) consisting of the ∼4,400 deletion mutants tested. We analyzed the three different ontologies: GO Process, GO Function and GO Component, with default *p*-value (*P* < 0.01) and false discovery rate filter thresholds.

### Yeast co-expression, immunoprecipitations and sample preparation

Yeast co-expression and immunoprecipitation was performed as described previously (Lewis *et al.* 2013). Briefly, overnight cultures of yeast strain Y7092 co-expressing FLAG-tagged HopZ1a (wild type or a catalytically-inactive mutant, C216A) with putative acetylation targets MKRP1 or HINKEL were diluted into fresh SD-Leu (2% raffinose) and allowed to grow at 30° for two doublings prior to inducing expression of effector and targets by addition of galactose to a final concentration of 2%. Following 15 h of induction, cultures were mechanically lysed and lysates were incubated with an anti-FLAG agarose resin (Sigma). The resin was washed in cell lysis buffer (50 mM Tris, pH = 8; 150 mM NaCl; 1.5 mM magnesium acetate; 5 mM EDTA; 0.15% NP-40) as described previously, with reduced NP-40 (0.015%) for the last of three washes (Lewis *et al.* 2013). After washing the resin to remove unbound proteins, FLAG-tagged proteins were eluted by incubating with 100 uL of FLAG peptide solution (150 ug/mL FLAG peptide in TBS) for one hour at 4°. Eluted material was dried to a pellet under vacuum and stored at -80° prior to subsequent mass spectrometry analysis. Dried protein samples were re-solubilized in 50 mM ammonium bicarbonate (pH 7.8) and then subjected to reduction with dithiothreitol at 56°, alkylation with iodoacetamide at room temperature, and overnight digestion with sequencing-grade trypsin (Promega, Madison, WI) at 37°. The enzymatic reactions were stopped with 3% formic acid, purified and concentrated with Pierce C18 Spin Columns (Thermo Scientific) and again dried to a pellet under vacuum. Peptide samples were then solubilized in 0.1% formic acid prior to LC-MS/MS analyses.

### LC-MS/MS Analysis of Proteins, Chromatography and Mass Spectrometry

Subsequent analytical separation was performed on a homemade gravity-packed 75 µm internal diameter column (New Objective, Woburn, MA) packed with 10 cm of 100 Å, 5 µm Magic C18AQ particles (Michrom, Auburn, CA). Peptide samples were loaded onto the analytical column using a variable gradient with a flow rate of 300 nL/min. The gradient utilized two mobile phase solutions: A, water/0.1% formic acid; and B, 80% acetonitrile/0.1% formic acid. Samples were analyzed on a linear ion trap-Orbitrap hybrid analyzer outfitted with a nano spray source and EASY-nLC 1200 nano-LC system. The instrument method consisted of one MS full scan (400–1400 *m*/*z*) in the Orbitrap mass analyzer, an automatic gain control target of 500,000 with a maximum ion injection of 500 ms, one microscan, and a resolution of 60,000. Six data-dependent MS/MS scans were performed in the linear ion trap using the three most intense ions at 35% normalized collision energy. The MS and MS/MS scans were obtained in parallel fashion. In MS/MS mode automatic gain control targets were 10,000 with a maximum ion injection time of 100 ms. A minimum ion intensity of 1000 was required to trigger an MS/MS spectrum. The dynamic exclusion was applied using an exclusion duration of 145s.

### Protein ID and Database Searching

Proteins were identified by searching all MS/MS spectra against a large database composed of the complete proteome of *Saccharomyces cerevisiae* strain S288C (ATCC 204508; UniProt proteome ID UP000002311) supplemented with sequences for *P. syringae* HopZ1a (WP_011152901.1), and the *Arabidopsis* kinesins HINKEL (NP_173273.2) and MKRP1 (NP_173592.3) (all retrieved from the NCBI database) using SEQUEST (Thermo Scientific Proteome Discoverer software). A fragment ion mass tolerance of 0.6 Da and a parent ion tolerance of 10 ppm were used. Up to two missed tryptic cleavages were allowed. Methionine oxidation (+15.99492 Da), cysteine carbamidomethylation (+57.02146 Da), and acetylation (+42.01057 Da) were set as variable modifications. The generated search results were imported into the Scaffold data analysis platform, an X!Tandem search (Beavis Informatics, Winnipeg, MA) was performed and the peptides were evaluated using a false discovery rate of 0.1% as determined using a reversed version of the database used in the original search. A mzident.xml file was generated from Scaffold and imported into Scaffold PTM (Proteome Software, Portland, OR) to evaluate and score the post translational modifications.

### Yeast strains and Data availability

**Figure 1:**
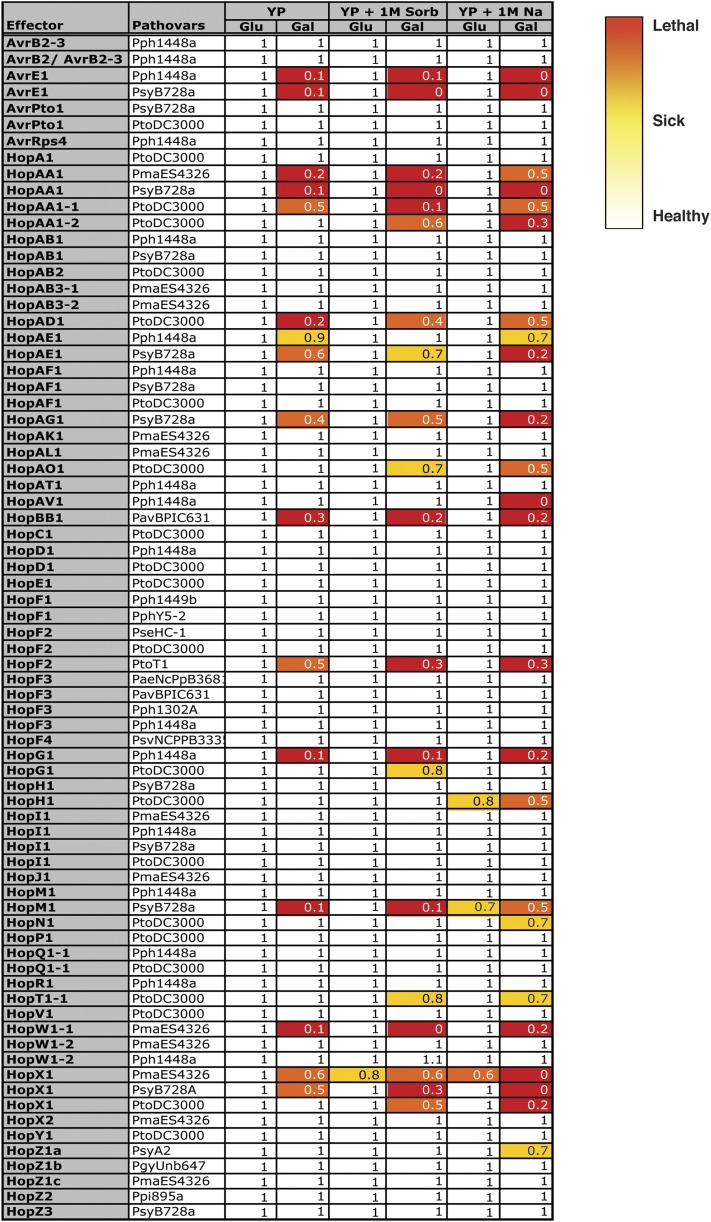
Growth inhibition profiles of yeast (Y7092) expressing 75 *P. syringae* T3SEs on rich media (Hong *et al.*), rich media with 1 M sorbitol (YP + 1M Sorb), and rich media with 1 M NaCl (YP + 1M Na). The growth inhibition by each T3SE in each condition is represented in numbers and heat map, with 1 (or white) corresponding to no growth inhibition to 0 (or red) corresponding to complete growth inhibition. The fitness numbers are calculated for every condition (Glu = glucose and T3SE-repressing, Gal = galactose and T3SE-expressing) by normalizing the fitness of yeast expressing T3SE to the negative control strain containing the integrated *NAT^R^* antibiotic cassette at the *ho* locus.

**Figure 2:**
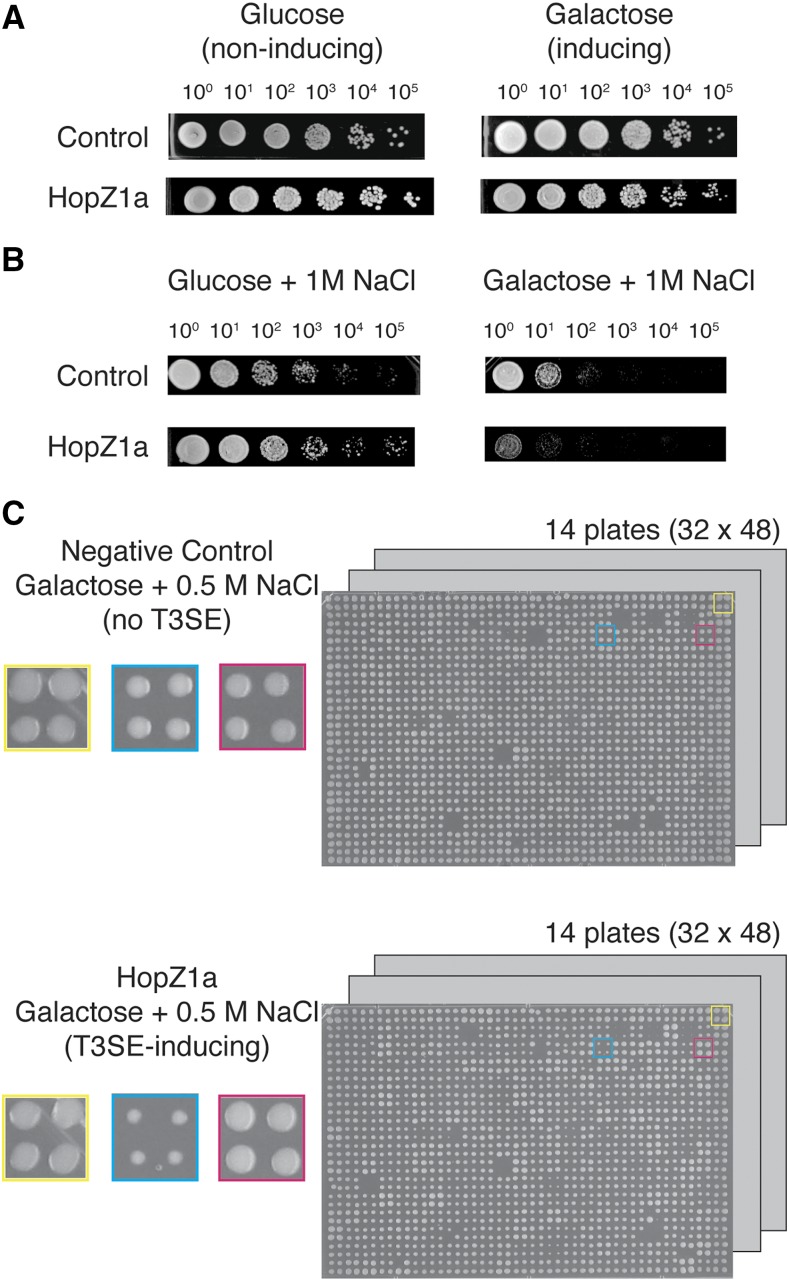
Genome-wide phenotypic screens to identify yeast deletion strains that suppressed or were sensitized to *P. syringae* T3SE expression. (A) HopZ1a does not inhibit yeast growth on rich media as shown by spot dilution assay. The control strain has the *NAT^R^* antibiotic cassette integrated at the *ho* locus. (B) The growth inhibition by HopZ1a compared to the negative control strain on rich media with 1M NaCl (galactose + 1M NaCl) by spot dilution assay is shown. (C) Yeast haploid deletion collection with the integrated *GAL1-hopZ1a* at the *ho* locus (*hoΔ*::*GAL1-hopZ1a-FLAG*::*NAT^R^*) on galactose (HopZ1a-inducing) media with 0.5 M NaCl. The negative control array was also pinned on the galactose media with 0.5 M NaCl. Each deletion mutant was pinned in quadruplicate onto the appropriate solid media to generate four replicates in each screen. Colonies in the yellow square represent the border control strain, colonies in the blue square represent a yeast deletion strain that is sensitive to HopZ1a expression, while colonies in the red square represent a yeast deletion strain that suppresses fitness defects as a result of HopZ1a expression.

**Figure 3:**
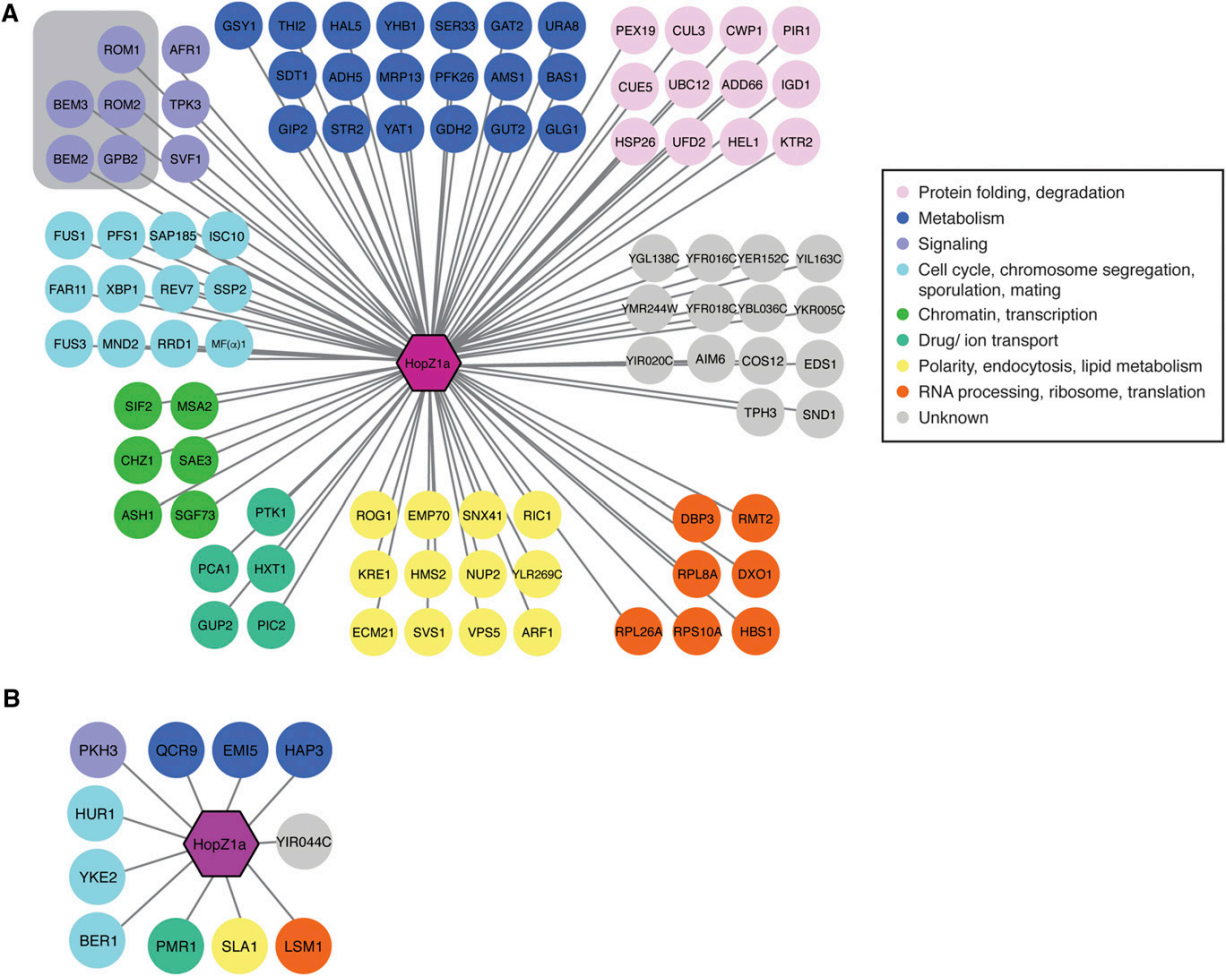
Analysis of HopZ1a suppressors and negative genetic interactors in yeast. Diagram showing (A) suppressors and (B) negative genetic interactors of HopZ1a generated using Cytoscape. Nodes are color coded based on annotations of biological processes from Constanzo *et al.* (Costanzo *et al.* 2010). The HopZ1a suppressors showed an enrichment in GTPase-mediated signal transduction (gray-shaded box; *P =* 0.006).

**Figure 4:**
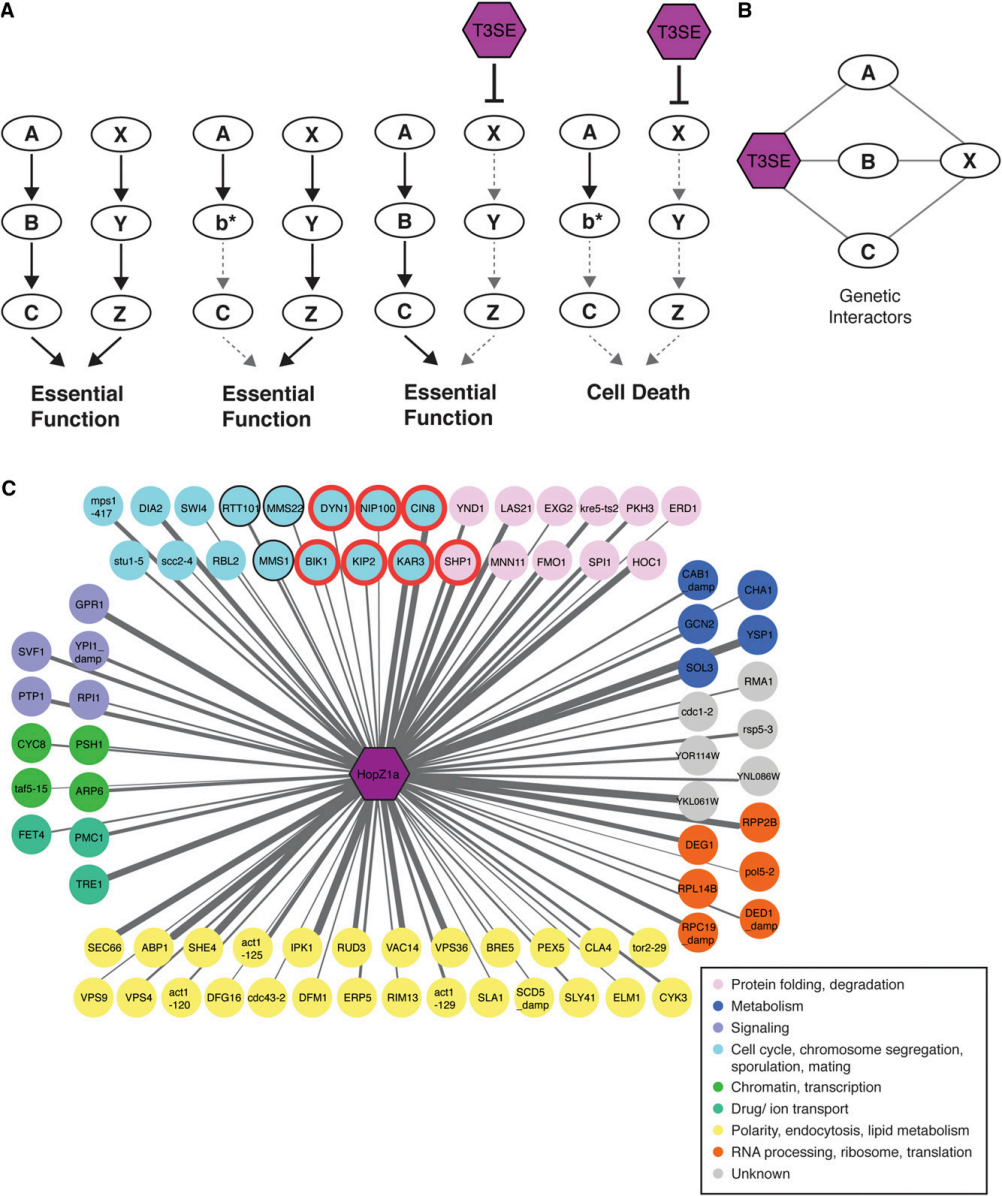
Congruence gene analyses of HopZ1a negative genetic interactors identify microtubule motor proteins as potential targets. (A) A model for the molecular mechanism of enhancing T3SE toxicity by targeting redundant pathways. A mutation in either one of the parallel redundant pathways (b* or the inhibition of X by T3SE) does not alter cell viability. However, when both pathways are disrupted (b* and the inhibition of X by T3SE), the cells are not viable. (B) Congruence analysis predicts potential T3SE targets by identifying yeast genes (gene X) with similar genetic interaction profiles as the T3SE. (C) 81 congruent yeast genes with congruence score ≥ 2 are shown, with nodes color coded based on annotations of biological processes from Constanzo *et al.* (Costanzo *et al.* 2010). HopZ1a is congruent to yeast deletion strains that are enriched for replication fork processing (*P* < 0.0001) and microtubule-based processes (with a *P* < 0.0004) as analyzed by GOrilla tool (Eden *et al.* 2009). Genes enriched in microtubule-based processes are circled in red, and genes enriched for replication fork processing are circled in black. Edge thickness is proportional to congruence scores.

**Figure 5:**
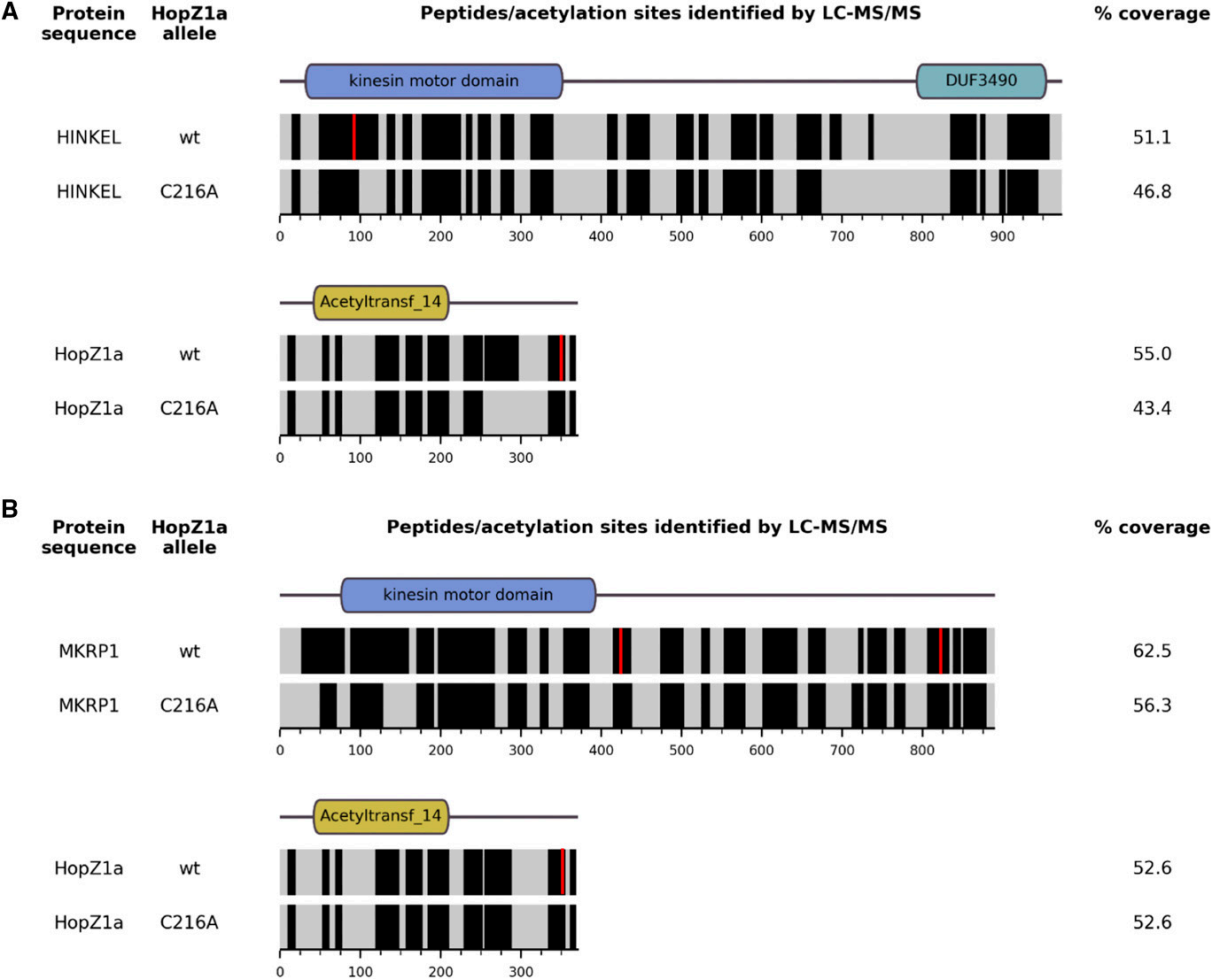
HopZ1a acetylates *A. thaliana* kinesins HINKEL and MKRP1. Predicted domain architectures (as annotated by the NCBI Conserved Domain Database; Marchler-Bauer *et al.* 2015) for HINKEL (A), MKRP1 (B) and HopZ1a (A and B) are indicated above horizontal bands representing the mass spectrometry sequence coverage for each protein. Black bands indicate sequences identified with high confidence, while gray bands indicate sequences that were not reliably detected. Red vertical stripes indicate the position of acetylated residues.

All yeast strains and plasmids described in this study are available upon request. Mass spectrometry data consisting of raw files and associated peak list and results files has been deposited in MassIVE as complete (Data Dependent Acquisition). Mass spectrometry data are available from MassIVE (https://massive.ucsd.edu) using Massive ID: MSV000083076. Table S1.xlsx: list and description of confirmed genetic interactions for HopZ1a. Table S2.xlsx: list and description of confirmed genetic interactions for HopF2. Table S3.xlsx: list and description of confirmed genetic interactions for HopX2. TableS4.xlsx: congruence scores for yeast genes with genetic interaction profiles similar to that of HopZ1a. TableS5.xlsx: congruence scores for yeast genes with genetic interaction profiles similar to that of HopF2. TableS6.xlsx: congruence scores for yeast genes with genetic interaction profiles similar to that of HopX1. FigureS1.tiff: immunoblot analysis of yeast strain Y7092 expressing *P. syringae* T3SEs. FigureS2.tiff: spot dilution assays to determine growth inhibition profiles of yeast expressing *P. syringae* T3SEs. FigureS3.pdf: extracted ion chromatograms, reversed phase chromatography and MS/MS spectra supporting identification of two distinct (singly) acetylated forms of the doubly charged HINKEL peptide, VFGPESLTENVYEDGVK. FigureS4.pdf: extracted ion chromatograms, reversed phase chromatography and MS/MS spectra supporting acetylation of the doubly and triply charged MKRP1 peptide, EISCLQEELTQLR. FigureS5.pdf: extracted ion chromatograms, reversed phase chromatography and MS/MS spectra supporting acetylation of the doubly and triply charged MKRP1 peptide, EIYNETALNSQALEIENLK. FigureS6.pdf: extracted ion chromatograms, reversed phase chromatography and MS/MS spectra supporting acetylation of the doubly and triply charged HopZ1a peptide, ELLDDETPSNTQFSASIDGFR. FigureS7.pdf: zoomed-in views of the extracted ion chromatograms presented in Figure S6. FigureS8.pdf: acetylated HINKEL residues are proximal to the kinesin ATP-binding site. Supplemental material available at Figshare: https://doi.org/10.25387/g3.7318505.

## Results

### Generation and characterization of yeast strains carrying P. syringae T3SEs

We generated a collection of 75 yeast strains each carrying an inducible *P. syringae* T3SE expression construct (Figure 1). The T3SEs included those from three widely studied *P. syringae* strains: 22 T3SEs from *P. syringae* pv. *tomato* DC3000 (*Pto*DC3000); 12 T3SEs from *P. syringae* pv. *syringae* B728a (*Psy*B728a); and 17 T3SEs from *P. syringae* pv. *phaseolicola* 1448A (*Pph*1448a). These three strains have finished genome sequences and represent three of the five major *P. syringae* phylogroups (phylogroups 1, 2, and 3, respectively) (Hwang *et al.* 2005). We also screened 12 T3SEs from *P. syringae* pv. *maculicola* ES4326 (*Pma*ES4326), which belongs to phylogroup 4. Finally, we screened three additional T3SEs from the HopZ family and nine additional T3SEs from the HopF family, as these two effector families are of particular interest to our group (Figure 1) (Ma *et al.* 2006; Lewis *et al.* 2008; Wilton *et al.* 2010). Briefly, each T3SE construct was linked to a drug resistance cassette (*NAT^R^*) and integrated at the *ho* locus – a neutral, dispensable locus not functionally required in stable haploid or diploid cells (Baganz *et al.* 1997; Youn *et al.* 2017). Each T3SE was tagged with a C-terminal FLAG epitope and expressed under the control of a galactose-inducible promoter. We confirmed galactose-dependent expression of the 75 T3SEs using western blot analysis (Figure S1).

Using the collection of T3SE-expressing yeast strains we performed a fitness-based screen to identify T3SEs that inhibit yeast growth. We examined the phenotypic consequence of T3SE expression in yeast using serial dilution spot assays on rich media with glucose (T3SE-repressing) or galactose (T3SE-expressing). As expected, we did not observe fitness defects on T3SE-repressing media (Figure 1 and S2) compared to the negative control strain (*hoΔ*::*NAT^R^*), however, the expression of 16 out of 75 T3SEs inhibited yeast growth on T3SE-expressing rich media (Figures 1 and S2: AvrE*_Pph1448a_*, AvrE*_PsyB728a_*_,_ HopAA1*_PmaES4326_*, HopAA1*_PsyB728a_*, HopAA1-1*_PtoDC3000_*, HopAD1*_PtoDC3000_*, HopAE1*_Pph1448a_*, HopAE1*_PsyB728a_*, HopAG1*_PsyB728a_*, HopBB1*_PavBPIC631_*, HopF2*_PtoT1_*, HopG1*_Pph1448a_*, HopM1*_PsyB728a_*, HopW1-1*_PmaES4326_*, HopX1*_PsyB728a_* and HopX1*_PmaES4326_*).

To identify additional T3SEs that may target conserved cellular processes under stress conditions, we also performed fitness assays on media inducing hyperosmotic stress (containing 1 M sorbitol or 1 M NaCl). Nine additional T3SEs altered yeast fitness when expressed in the presence of high osmolytes, with yeast expressing HopW1-2*_Pph1448a_* showed a slightly increased fitness on 1 M sorbitol (Figures 1 and S2). Four of the *Pto*DC3000 T3SEs caused enhanced fitness defects in yeast both with 1 M sorbitol and with 1 M NaCl (HopAA1-2, HopAO1, HopT1-1, and HopX1). Although 1 M sorbitol and 1 M NaCl both activate the high osmolarity glycerol (Hohmann 2002) pathway by creating a high osmolarity environment, NaCl stress creates additional toxicity by altering the ion homeostasis in the cell (Giaever *et al.* 2002). We also identified a single T3SE that affected yeast fitness only in the presence of 1 M sorbitol (HopG1*_PtoDC3000_*) and three T3SEs that altered yeast fitness only in the presence of 1 M NaCl (HopAV1*_Pph1448a_*, HopN1*_PtoDC3000_* and HopZ1a*_PsyA2_*).

### Identifying genetic interactors of P. syringae T3SEs by PGA analysis

To further characterize *P. syringae* T3SE functions and their mechanisms of toxicity in yeast, we utilized the yeast PGA functional genomics approach on HopAA1, HopW1-1, HopZ1a, HopF2 and HopX1. To this end, we performed a PGA screen by crossing our integrated T3SE-expressing strain with ∼4400 haploid yeast non-essential gene deletion mutants (Giaever *et al.* 2002), looking for negative and positive genetic interactions in the context of T3SE expression (Figure 2C). We carried out a parallel control screen using a query strain (Youn *et al.* 2017) harboring a deletion in a benign locus (*ho)* instead of a T3SE (see Materials and Methods section for details) to obtain ‘control arrays’ that reflect the fitness of the yeast gene deletion mutants.

We manually scored positive or negative genetic interactions by observing changes in colony size (fitness) between the experimental and control plate (Figure 2C; see Materials and Methods for further details). We classified negative genetic interactors for those double mutants that grew more poorly than expected based on those of the single mutant fitness. In addition, if the double mutant grew much better than that of a single mutant (in this case, T3SE), we classified these interactions as suppression.

We observed potential genetic interactors in the PGA screens of HopF2 (132 suppressors and 73 synthetic lethal interactors) and HopX1 (88 synthetic lethal interactors), whereas HopAA1 and HopW1-1, with the most severe fitness defect, did not reveal any reproducible interactors (including suppressors) in our initial PGA analysis. As for HopZ1a, we observed no genetic interactions under standard PGA conditions, which prompted us to test genetic interactions in a condition that shows HopZ1a-induced fitness defect: high osmotic stress condition. Since 1 M NaCl drastically reduced the fitness of the HopZ1a-expressing yeast strain, we therefore assessed the fitness of ∼4400 double mutants on media containing a range of salt concentrations below 1 M NaCl. At 0.25 M and 0.5 M NaCl, we initially identified 137 deletion mutants with reduced HopZ1a toxicity (suppressors) and 53 deletion mutants with enhanced HopZ1a toxicity (negative genetic interactors; data not shown).

To confirm the genetic interaction phenotypes, we conducted a secondary screen by transforming the haploid yeast deletion strains that were identified in our primary PGA screen with single copy plasmid (p*BA350V)* carrying *GAL-T3SE* (HopZ1a, HopF2 or HopX1), and then used spot dilution assays to characterize fitness. We excluded any strains with deletions in dubious open reading frames (Winzeler *et al.* 1999; Giaever *et al.* 2002; Kramer *et al.* 2007) as well as galactose metabolism genes. For HopZ1a, 95 suppressors and 10 negative genetic interactors were confirmed by independent transformation and spot dilution assays on 0.5 M NaCl and galactose (Figure 3 and Table S1). For HopF2, 105 suppressors and 20 negative interactions were confirmed (Table S2), whereas 32 negative interactions (no suppressors) were confirmed for HopX1 (Table S3). Confirmed genetic interactors for HopZ1a, HopF2 and HopX1 can be found in Supplementary Tables S1-S3.

### Biological processes enriched in PGA profiles

We utilized the Gene Ontology (GO) vocabulary to identify biological processes associated with the confirmed HopF2, HopX1 and HopZ1a PGA interaction partners, since GO processes that are enriched within this genetic interaction data set may potentially illuminate functional processes that are influenced by effectors (Kramer *et al.* 2007; Baryshnikova *et al.* 2010). We analyzed positive and negative interactors separately.

Using the *Saccharomyces* Genome Database (SGD) GO Term Finder (Hong *et al.* 2008), we found a significant enrichment of genes involved in GTPase-mediated signal transduction and its regulation (Figure 3A; *P* = 0.006) in HopZ1a suppressors. Specifically, we identified two Rho GTPase activating proteins that are critical for cell polarity and cell division: BEM2 and BEM3; as well as two GDP/GTP exchange proteins: ROM1 and ROM2. In contrast, no significant GO enrichment was found for HopF2 suppressors (no HopX1 suppressors were identified).

Negative genetic interactors of HopF2 were enriched for various intracellular trafficking pathways (endosomal transport *P* < 0.004, vacuolar transport, *P* < 0.01, late endosome to vacuole transport via multi vesicular body sorting pathway, *P* < 0.06), whereas HopX1 negative interactors were enriched for protein complex assembly and biogenesis (*P* < 0.01), lipid tube assembly (*P* < 0.02), protein-lipid complex assembly (*P* < 0.02), mitochondrial respiratory chain complex IV assembly (*P* < 0.06). We did not identify significant enrichment of GO processes in the HopZ1a negative genetic interactors. However, two negative genetic interactors of HopZ1a, YKE2 and BER1, are involved in regulating tubulin folding and microtubule-related processes (Figure 3B). Additionally, we identified both suppressors (BEM2, BEM3 and RRD1) and negative genetic interactors (SLA1) that are involved in regulating the actin cytoskeleton (Figure 3A and B). Actin and microtubule cytoskeletons are both involved in fundamental processes such as cell division and intracellular trafficking, raising the possibility that our genetic interaction screen identified genes whose functions influence both of these two important cytoskeletal components.

### Predicting HopZ1a targets by congruence analysis of genetic interactors

Previously we have shown that the T3SE HopZ1a can bind to tubulin and alter microtubule networks *in planta* (Lee *et al.* 2012). We were particularly interested in the role of HopZ1a in regulating microtubule dynamics (and potentially other processes) and we therefore focused our analysis on this effector as proof-of-principle that our genomic resource can be used to characterize *P. syringae* T3SE functions.

The analysis of the HopZ1a genetic interactors described above revealed several biological processes that may be disrupted by HopZ1a but provided limited insight regarding its direct targets. We therefore sought to predict direct targets by identifying yeast gene disruptions that show similar (*i.e.*, congruent) genetic interaction profiles to HopZ1a. This approach is similar to one used previously to identify drug targets in yeast (Costanzo *et al.* 2010) and assumes that if HopZ1a activity disrupts a given target protein’s function in yeast, the HopZ1a PGA profile would be similar (or ‘congruent’) to the SGA profile of the corresponding gene knockout strain lacking this putative target (Figure 4A and B). We focused our congruency analyses on negative genetic interactors since previous work has indicated that these interactions are easier to interpret than suppressors (Ye *et al.* 2005).

To identify yeast genes with HopZ1a-congruent genetic interaction profiles, we compared our HopZ1a genetic interaction profile with those of 1712 single yeast mutants (encompassing ∼170,000 interactions) and calculated the pairwise overlap of genetic interactions (Costanzo *et al.* 2010) using a previously established congruence score (Ye *et al.* 2005). In brief, congruence score is defined as the –log_10_ of the *p*-value for the number of shared genetic interaction profiles of two genes and provides a ranking of the degree of similarity in genetic interaction profiles. Therefore, for any particular congruent gene pair, the overlap in shared genetic interacting partners increases with increasing congruence score (Table S4 – S6). We identified 81 yeast genes with HopZ1a congruence scores ≥ 2, indicating similarity to the negative genetic interaction profile of HopZ1a (Figure 4C and Table S4). We performed GO biological process enrichment analysis on this set of yeast genes (those with interaction profiles congruent to HopZ1a), and we found significant GO enrichment for genes involved in replication fork processing (*P* < 0.0001) (Figure 4C; circled black) and for genes involved in microtubule-based processes (*P* < 0.0004) (Figure 4C; circled red). These GO enrichment categories were specific to HopZ1a since HopF2 congruent genes were enriched for vesicle-mediated transport (*P* < 0.08; Table S5) whereas those of HopX1 were enriched for cell cycle (*P* < 0.0005), mitotic cell cycle process (*P* < 0.02), double-strand break repair via homologous recombination (*P* < 0.07; Table S6; data not shown).

Given that HopZ1a has been shown to disrupt microtubule networks in *Arabidopsis*, we were particularly interested in the congruent genes involved in microtubule-based processes (*P* < 0.0004). These included several microtubule-directed motor proteins such as kinesins (*i.e.*, CIN8, KIP2, VIK1, and KAR3), which have been shown to be HopZ-interacting proteins in two published yeast two-hybrid datasets between *A. thaliana* genes and *P. syringae* T3SEs (Mukhtar *et al.* 2011; Lewis *et al.* 2012). This overlap between the genetic and physical interactions observed for HopZ1a motivated further investigation into whether *Arabidopsis* kinesins represent direct targets of HopZ1a activity.

### HopZ1a acetylates plant kinesins

Kinesins are microtubule-based motor proteins involved in many cellular processes, including intracellular transport, mitotic cell division, signaling, and microtubule organization (Zhu and Dixit 2012). There are 61 kinesins in *Arabidopsis* (Lee and Liu 2004) and nearly one quarter of these (15) are members of the kinesin 7 (Kin7) subfamily, which were shown to interact with the HopZ family (Richardson *et al.* 2006; Mukhtar *et al.* 2011; Lewis *et al.* 2012). Given the large number of potential targets, we focused on kinesins that 1) interact with the HopZ family and 2) related kinesins that have been demonstrated to regulate plant microtubule stability. The kinesins previously shown to interact with the HopZ family are the mitochondrially-localized MKRP1 (At1g21730) and MKRP2 (At4g39050) (Mukhtar *et al.* 2011; Lewis *et al.* 2012). Since mitochondrial localization of HopZ1a has not been observed (Lewis *et al.* 2008), we also investigated whether HopZ1a may target the related Kin7 kinesin HINKEL (also known as AtNACK1 or HIK), which is involved in regulating microtubule stability in plants (Strompen *et al.* 2002; Takahashi *et al.* 2010; Komis *et al.* 2011).

HopZ1a is an acetyltransferase with multiple eukaryotic targets, including tubulin and the *A. thaliana* pseudokinase ZED1 (Lee *et al.* 2012; Lewis *et al.* 2013). To test whether HopZ1a acetylates kinesins *in vivo* in a heterologous yeast system, we co-expressed HopZ1a in yeast with each candidate kinesin, all as FLAG-tagged recombinant proteins, as previously described for *Arabidopsis* pseudokinase, ZED1 (Lewis *et al.* 2013). We used liquid chromatography tandem mass spectrometry (LC-MS/MS) to identify acetylated peptides of both HINKEL and MKRP1. LC-MS/MS analysis of anti-FLAG immunoprecipitates identified acetylated peptides from both kinesins (mass increases in multiples of 42 Daltons) present when co-expressed with wild type HopZ1a but not with the catalytically inactive mutant, HopZ1a^C216A^ (Figure 5). Candidate acetylation sites were confirmed by manual inspection of extracted ion chromatograms and MS/MS spectra (Figures S3-S7 and data not shown). In this way we identified two distinct acetylated species of the same HINKEL peptide (VFGPESLTENVYEDGVK; residues 83-99) - ‘peptide A’ (VFGPE[S-Ac]LTENVYEDGVK, acetylated at S88) and ‘peptide B’ (VFGPESL[T-Ac]ENVYEDGVK, acetylated at T90) (Figures 5A, S3). We also identified acetylated peptides from two distinct sites in MKRP1 - ‘peptide C’ (EISCLQEEL[T-Ac]QLR; residues 416-428; acetylated at T425) and ‘peptide D’ (EIYNE[T-Ac]ALNSQALEIENLK; residues 815-33; acetylated at T820) (Figures 5B, S4 and S5). Similar analysis of the HopZ1a-derived peptides from those cells co-expressing MKRP1 or HINKEL indicates auto-acetylation of HopZ1a at three sites in close proximity (T342, S344, T346) (Figures 5, S6, S7), consistent with a recent report that also described auto-acetylation of T346 (Ma *et al.* 2015).

## Discussion

In this study, we report the generation of a yeast strain collection stably expressing T3SEs from the plant pathogen *P. syringae* and demonstrate its utility for functional characterization of T3SEs. Out of 75 *P. syringae* T3SEs in yeast, we identified 24 effectors that altered yeast fitness on rich media or under high osmolarity conditions, including HopZ1a. Using HopZ1a as proof-of-principle, we took advantage of the genetic tractability of yeast to perform a high-throughput PGA screen to look for conserved biological processes that are targeted by HopZ1a. Exploiting a large genetic interaction dataset that covers ∼75% of all yeast genes, we performed congruency analysis to delineate conserved targets of HopZ1a in yeast and combined this with previously-described physical interaction datasets that include HopZ family members to implicate kinesins as potential targets of the T3SE, HopZ1a.

Previous studies have identified bacterial phytopathogen T3SEs that altered yeast fitness (Jamir *et al.* 2004; Munkvold *et al.* 2008; Salomon *et al.* 2011). Of the 27 *Pto*DC3000 effectors tested by Munkvold *et al.* 7 inhibited yeast growth (Munkvold *et al.* 2008; Munkvold *et al.* 2009). We tested 20 of these same 27 *Pto*DC3000 effectors and observed fitness phenotypes consistent with these previous data in all cases except for HopAO1, HopD1 and HopN1 (Munkvold *et al.* 2008; Munkvold *et al.* 2009). While we integrated T3SEs into the yeast genome and expressed them as single copy genes, Munkvold *et al.* expressed T3SEs on a high-copy plasmid. Differences in gene dosage may have contributed to these differences.

Our initial screen provides numerous interesting leads for further study. Notably, *P. syringae* T3SEs encoded in the conserved effector locus (CEL) caused severe fitness defects in yeast (Figure 1). T3SEs of the CEL are conserved across most *P. syringae* strains and typically include the evolutionarily unrelated T3SEs AvrE, HopM1, and HopAA1 (Alfano *et al.* 2000). *Pph*1448a has nonfunctional alleles of HopM1 and HopAA1 (Joardar *et al.* 2005), while *Pto*DC3000 contains an additional effector in its CEL, HopN1 (O’Brien *et al.* 2011). The CEL has been shown to play an important role in bacterial virulence (Alfano *et al.* 2000; Badel *et al.* 2003; Munkvold *et al.* 2009) and in the suppression of salicylic acid (SA)-mediated basal immunity (DebRoy *et al.* 2004). However, with the exception of HopM1 (Nomura *et al.* 2006; Nomura *et al.* 2011), the host targets and the mechanisms by which T3SEs in the CEL promote virulence are not well characterized. Our results suggest that most CEL T3SEs may have evolved to target conserved components of eukaryotic processes. The yeast fitness defects induced by expression of CEL T3SEs observed in this study will provide an important tool to help identify virulence targets of this ubiquitous class of phytopathogen T3SEs.

The PGA approach can be used to infer the function of T3SEs by identifying those yeast genes whose deletions either suppress or enhance T3SE lethality. Intuitively, deletion strains that suppress T3SE lethality (known as suppressors) can reveal genes involved in the same pathways as putative T3SE targets. This can be particularly informative when the T3SE activates a pathway resulting in toxicity, as was observed with the *Shigella* T3SE IpgB2 which activates the Rho1p GTPase signaling pathway in yeast (Alto *et al.* 2006). However, one caveat of the suppressor screen is that we may identify mutants that suppress T3SE lethality by a general mechanism (*i.e.*, by induction of a general stress response); such genes are unlikely to be informative for the inference of T3SE function.

Deletion mutants that exacerbate the fitness cost of T3SE activity can be explained by either of two alternate mechanisms resulting in ‘negative genetic interactions’. In one case, the T3SE acts in the same pathway as the ‘negative genetic interactor’, resulting in cumulative insults to an essential pathway or complex (Boone *et al.* 2007; Dixon *et al.* 2009). Alternatively, the T3SE and ‘negative genetic interactor’ may act on parallel pathways, which redundantly contribute to an essential function (Figure 3A) (Boone *et al.* 2007; Dixon *et al.* 2009). Our analysis of both suppressors and negative genetic interactors revealed enrichment of signal transduction pathways involving small-GTPases and may reflect an ability of HopZ1a to influence these cellular processes. Similarly, HopF2 and HopX1 may influence cellular trafficking and lipid metabolism, respectively. In order to gain further insight into the direct targets of HopZ1a we applied a congruence analysis to compare SGA interaction profiles of 1,712 yeast genes, including 334 conditional alleles of essential genes (Costanzo *et al.* 2010) with the HopZ1a PGA interaction profile described in this study. This approach is conceptually similar to the integration of chemical-genetic and SGA datasets for identification of drug targets (Costanzo *et al.* 2010); functional inhibition of a target protein by drug or by T3SE is expected to mimic the consequences of the corresponding target gene’s deletion, resulting in similar/congruent genetic interaction profiles.

Applying these principles, we identified SGA profiles that were most similar to the HopZ1a PGA profile and analyzed them for functional enrichment. Genes involved in replication fork processing (*P* < 0.0001) and microtubule-based processes (*P* < 0.0004) were enriched in the subset with HopZ1a-congruent interaction profiles. We were particularly interested in microtubule-associated processes since HopZ1a can disrupt microtubules in plants and interacts with tubulin in both plant and animal cells (Lee *et al.* 2012). Indeed, kinesins (known microtubule-guided motor proteins) were identified not only through our congruence analysis, but also by two independent yeast two-hybrid screens for *Arabidopsis* proteins that bind to related HopZ family members. The fact that kinesins are found at the intersection of these three independent datasets indicates that members of this family may indeed represent *bona fide*, direct targets of HopZ1a. In support of this possibility, HopZ1a can acetylate both of the *Arabidopsis* kinesins HINKEL and MKRP1 (Figure 5).

The acetylated sites (S88, T90) of HINKEL are found within its kinesin motor domain (Figure 5A), and mapping these to the corresponding positions in the structure of human kinesin CENP-E (Garcia-Saez *et al.* 2004) reveals a close proximity to the nucleotide-binding pocket (Figure S8). In *A. thaliana*, HINKEL activates the ANP1/ANQ1/MPK4 MAPK pathway that ultimately regulates microtubule-bundling proteins (*e.g.*, MAP65) via phosphorylation (Komis *et al.* 2011). Our data suggest a possible mechanism for HopZ1a-mediated antagonism of this pathway whereby nucleotide binding and/or hydrolysis activity is altered following acetylation of sites proximal to the nucleotide-binding pocket of HINKEL.

Although HopZ1a has not been detected in mitochondria, we cannot rule out the possibility that the mitochondrial kinesins identified by yeast two-hybrid assays are also targeted by HopZ1a, especially considering that they are targeted by the *P. syringae* T3SE HopG1 and are involved in plant immunity (Shimono *et al.* 2016). HopZ1a acetylates MKRP1 at two distinct sites: T425 is just ‘downstream’ of the kinesin motor domain while T820 is near its C-terminus (Figure 5B). In *Nicotiana*, the HINKEL ortholog NACK1 is phosphorylated near the C-terminus at residues T675, T690 and T836 by cyclin-dependent kinases to regulate microtubule dynamics during cytokinesis (Sasabe *et al.* 2011). Although reasonable speculation might suggest that C-terminal acetylation could disrupt hypothetical phosphorylation sites of MKRP1 and other kinesins, MKRP1 however lacks the C-terminal DUF3490 domain common to HINKEL and NACK1 (Figure 5) and we did not detect HopZ1a acetylation at the C-terminus of HINKEL.

Additional acetylation sites may exist on HINKEL and MKRP1 (and HopZ1a) since LC-MS/MS analysis is unable to detect all peptides generated from trypsin digests of the proteins of interest; we only acquired 47–51% coverage of HINKEL, 56–63% coverage of MKRP1, and 43–55% coverage of HopZ1a (Figure 5). Thus, our acetylation analysis is conservative and it remains possible that HopZ1a acetylates additional residues of HINKEL and/or MKRP1 that we were unable to observe. Although HINKEL is acetylated within its kinesin motor domain at positions S88 and T90, the corresponding residues were not acetylated in MKRP1. The acetylation sites of MKRP1 are not present in HINKEL (not shown) and HINKEL has a C-terminal DUF3490 domain that is absent from MKRP1 (Figure 5). Thus, if acetylation of these two kinesins is an important function of HopZ1a *in planta*, they are likely to be regulated by contrasting mechanisms. Nevertheless, these data indicate that HopZ1a can target *A. thaliana* Kinesin 7 family members.

Overall, we believe that the library of T3SE-expressing yeast strains developed in this study represents a powerful resource to functionally characterize T3SE from *P. syringae*.
